# Posture-Dependent Focal Neurologic Deficits in Meningioma-Related Superior Sagittal Sinus Obstruction: An Illustrative Case

**DOI:** 10.7759/cureus.107042

**Published:** 2026-04-14

**Authors:** Ugoma Onubogu, Victor Milev, Sinjana Kolipaka, Stephen A Zorc, Peter Averkiou

**Affiliations:** 1 Department of Medicine, Florida Atlantic University Charles E. Schmidt College of Medicine, Boca Raton, USA; 2 Department of Integrated Medical Science, Florida Atlantic University Charles E. Schmidt College of Medicine, Boca Raton, USA; 3 Initiative for Maximizing Student Development (IMSD), Florida Atlantic University Charles E. Schmidt College of Medicine, Boca Raton, USA

**Keywords:** neuro-oncology, positional symptom modulation, superior sagittal sinus compression, venous hypertension, venous stasis

## Abstract

Venous hypertension contributes to a broad range of clinical manifestations across organ systems. In many venous disorders, symptoms fluctuate with posture because gravitational and mechanical factors alter venous return and regional venous pressures. We present a unique case of posture-dependent focal neurologic deficits in a patient with a large malignant meningioma involving the superior sagittal sinus. The patient experienced recurrent transient right-sided weakness and paresthesia when reclined, with improvement after assuming an upright position. Neuroimaging demonstrated a large posterior vertex meningioma with the encasement of the superior sagittal sinus and no evidence of significant arterial occlusion. Published literature on meningioma-related venous sinus compromise primarily describes headache, papilledema, and elevated intracranial pressure rather than posture-dependent focal neurologic deficits. Although there is a physiologic rationale to suspect that positional change could influence symptoms in venous outflow disorders, this question remains largely unstudied in patients with meningioma-induced venous hypertension. Current evidence does not support positional change as an established disease-specific treatment in cerebrovascular venous disorders. We therefore present this case as a hypothesis-generating example of posture-sensitive venous pathophysiology in the intracranial compartment. The greater recognition of this phenomenon may improve diagnostic reasoning and inform future study of dynamic venous disorders.

## Introduction

Venous stasis describes a state of blood pooling due to impaired venous return and is a key contributor to venous hypertension and associated symptoms such as swelling, pain, and inflammation [1,2]. Across venous disorders, sustained venous hypertension contributes to edema, pain, inflammation, endothelial dysfunction, and, in some settings, secondary ischemic or neuropathic symptoms [1,3]. Because venous return is strongly influenced by gravity, body position, and local mechanical constraints, symptom severity in venous disease often fluctuates with posture or with maneuvers that alter hydrostatic pressure and venous outflow resistance [4-6].

This principle is also relevant to the intracranial venous system. The superior sagittal sinus is a major dural venous channel that receives drainage from superficial cortical and bridging veins arising from the superior and medial aspects of the frontal and parietal lobes, as well as portions of the occipital convexity [7]. These parasagittal frontal-parietal regions include cortical areas involved in contralateral motor and sensory function, and the involvement of dominant frontal networks may also contribute to speech disturbance [8]. Accordingly, the compromise of the superior sagittal sinus may impair venous drainage from functionally eloquent parasagittal cortex and produce transient neurologic symptoms through venous hypertension and secondary microcirculatory dysfunction [5,9].

Here, we describe a patient with a large posterior vertex meningioma involving the superior sagittal sinus who developed recurrent right-sided arm and leg weakness and paresthesia with intermittent word-finding difficulty and impaired enunciation, specifically when supine or reclined, with improvement after standing or ambulating. This reproducible positional pattern highlights a clinically recognizable form of posture-dependent symptom modulation in cerebral venous outflow obstruction. We present this case as a descriptive example of how body position may influence symptoms in selected intracranial venous disorders. The content of this article was presented as a meeting abstract at the 2025 Palm Beach County Medical Society Poster Symposium on September 13, 2025.

## Case presentation

The patient is a right-handed man in his early 80s with a past medical history notable for meningioma status post proton beam therapy, dyslipidemia, and hypertension, who presented to the emergency department as a stroke alert due to complaints of right-sided hemiparesthesia.

He began with right arm weakness the evening prior while he was reclining in a chair, followed by right leg weakness several minutes later. The patient went to sleep that night but awoke with worsened right arm weakness, prompting him to call emergency medical services. While en route to the emergency department on a stretcher, he noted the development of numbness in his right leg. He reported that the weakness had resolved by the time he arrived in the emergency department. The patient described six similar episodes over the past few months, several of which resulted in falls. Each episode was characterized by right-sided weakness and paresthesia occurring while lying down to sleep, upon waking, or while reclined, with spontaneous resolution after standing or walking. He also endorsed intermittent word-finding difficulty and mild dysarthric speech during episodes. He denied fever, chest pain, shortness of breath, headache, dizziness, vision changes, nausea, or vomiting. At the time of assessment, the patient was assigned a National Institutes of Health (NIH) Stroke Scale score of 1 for decreased sensation in the right upper extremity, and his symptoms fully resolved shortly after admission to telemetry.

**Figure 1:**
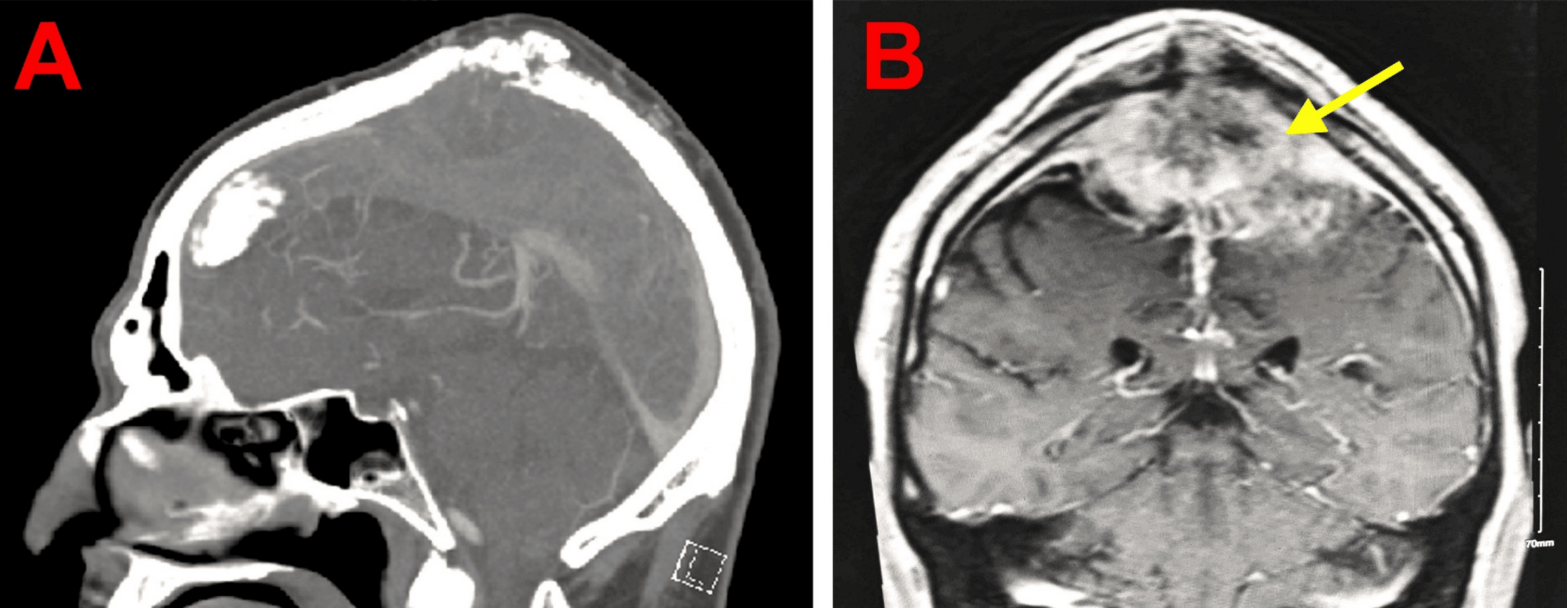
Neuroimaging of large meningioma involving the superior sagittal sinus (A) Non-contrast CT of the head showing a stable, large posterior vertex meningioma with no acute ischemia. (B) Brain MRI showing homogeneous enhancement, diffusion restriction, and superior sagittal sinus encasement CT, computed tomography; MRI, magnetic resonance imaging

The patient had been diagnosed with meningioma 20 years earlier and was followed by a neuro-oncologist. The surgical resection of the lesion had not been pursued due to its suboptimal location. He completed daily proton beam therapy for six weeks, finishing his regimen eight months prior to his presentation to the emergency department. Non-contrast computed tomography of the head demonstrated a stable, large posterior vertex meningioma but no evidence of acute ischemia (Figure 1A). Magnetic resonance imaging re-demonstrated the large posterior vertex meningioma with diffusion restriction, homogeneous enhancement, and the encasement of the superior sagittal sinus, raising concern for sinus occlusion (Figure 1B). The magnetic resonance angiography of the head showed no significant arterial stenosis or occlusion. An EKG showed a normal sinus rhythm.

## Discussion

**Figure 2:**
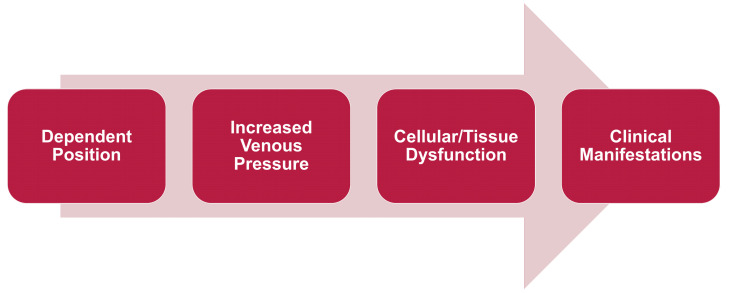
Chronology of position-dependent symptom exacerbation

This case is notable for recurrent, transient, focal neurologic deficits that were reproducibly provoked by the supine or reclined position and relieved by returning upright. In the context of a large posterior vertex meningioma encasing the superior sagittal sinus and the absence of major arterial stenosis or occlusion on vascular imaging, a plausible explanation is dynamic cerebral venous outflow obstruction with posture-sensitive venous hypertension (Figure 2).

Literature supports only part of this framework. Reports of meningioma-related venous sinus compromise primarily describe headache, papilledema, and elevated intracranial pressure rather than posture-dependent focal neurologic deficits [10,11]. Similarly, clinical series of meningioma-induced venous sinus stenosis have focused on pseudotumor-like presentations and procedural outcomes, including improvement in headache and papilledema after intervention, without characterizing whether symptoms varied according to body position [10,11]. Thus, while meningioma-related venous outflow compromise is recognized, the specific posture-linked focal neurologic pattern observed in this case appears to be poorly described. This gap is notable because the physiologic rationale for positional symptom modulation is strong. Head and body position influences venous pressures and intracranial pressure, and cerebral venous drainage varies with posture [4,5]. In patients with substantial superior sagittal sinus compromise, these normal shifts in venous return may become clinically consequential. A possible explanation in this case is that recumbency increased venous congestion within an already impaired drainage network, worsening venous hypertension, interstitial edema, and secondary microcirculatory compromise in adjacent cortical tissue. This interpretation also helps reconcile the transient ischemia-like character of the episodes with the absence of major arterial occlusion. Whether due to impaired arterial inflow or impaired venous outflow, focal neurologic deficits may emerge when local substrate delivery becomes inadequate [9]. In cerebrovascular disease, elevated venous pressure may reduce the effective arteriovenous pressure gradient, impair capillary exchange, and promote tissue edema, ultimately producing reversible neuronal dysfunction [1,9].

At the same time, systematic evidence supporting postural interventions in cerebrovascular venous disease remains extremely limited. In cerebral venous sinus thrombosis and related cerebrovascular venous disorders, published guidelines emphasize anticoagulation, selected endovascular intervention, and the management of intracranial hypertension rather than positioning as a disease-specific therapeutic strategy [9,12]. Although head-of-bed elevation is sometimes used as supportive management in patients with markedly elevated intracranial pressure, this does not constitute evidence for posture as a targeted treatment for venous outflow pathology itself [12,13]. Given the paucity of literature and comparable reported cases, this case is best understood as a hypothesis-generating example of posture-sensitive intracranial venous pathophysiology rather than evidence for a distinct syndrome or validated postural treatment strategy.

**Figure 3:**
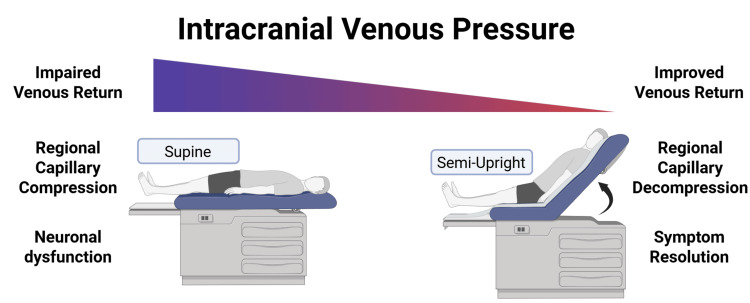
Graphical summary

The clinical implication remains important. In patients with known or suspected dural venous sinus involvement by tumor, recurrent neurologic symptoms that occur preferentially when supine, reclined, or otherwise positionally stressed should prompt the consideration of dynamic venous pathophysiology in addition to more familiar arterial and epileptic explanations. In this patient, the transient deficits likely arose from impaired cerebral venous drainage with consequent venous hypertension, tissue edema, and the secondary compression of cortical microcirculation, as summarized in Figure 3. The recognition of similar presentations may help guide future hemodynamic and positional study, including formal positional imaging, venography, venous manometry, or systematic symptom assessment.

## Conclusions

In summary, we report a case of a large malignant meningioma involving the superior sagittal sinus that manifested as recurrent focal neurologic deficits preferentially in the prolonged supine position, with symptom resolution after returning to the upright position. The case supports the possibility that cerebral venous outflow obstruction may, in selected patients, produce dynamic and posture-dependent neurologic symptoms. This presentation may reflect an underrecognized feature of intracranial venous outflow compromise and warrants further study with formal hemodynamic and positional assessment.
